# COVID-19 Impact on Chronic Myeloid Leukemia Patients

**DOI:** 10.3390/jpm12111886

**Published:** 2022-11-10

**Authors:** Dana Raluca Arbore, Simona Maria Galdean, Delia Dima, Ioana Rus, David Kegyes, Raluca Geanina Ababei, Daniela Dragancea, Radu Andrei Tomai, Adrian Pavel Trifa, Ciprian Tomuleasa

**Affiliations:** 1 Department of Hematology, Iuliu Hațieganu University of Medicine and Pharmacy, 400012 Cluj-Napoca, Romania; 2 Department of Hematology, Ion Chiricuta Clinical Cancer Center, 400012 Cluj-Napoca, Romania; 3 MEDFUTURE Research Center for Advanced Medicine, Iuliu Hatieganu University of Medicine and Pharmacy, 400012 Cluj-Napoca, Romania

**Keywords:** chronic myeloid leukemia, SARS-Cov-2, COVID-19, tyrosine kinase inhibitors, COVID-19 vaccine

## Abstract

(1) Background: Chronic myeloid leukemia (CML) is a blood dyscrasia that accounts for about 20% of all leukemia cases. Tyrosine kinase inhibitors (TKIs) are used as first line treatment of CML. The 2019 SARS-CoV-2 outbreak raised new concerns for CML patients, such as whether CML increases the risk of contracting COVID-19, whether TKIs increase that risk, whether these drugs are safe to use during the infection, and whether any other hematologic parameters influence infection outcomes. (2) Methods: In our study we addressed these intriguing questions by using a retrospective analysis of 51 CML patients treated at the Ion Chiricuta Cancer Center, Cluj-Napoca, Romania. Furthermore, we investigated the effects of currently approved COVID-19 vaccines in our CML patients treated with tyrosine kinase inhibitors. (3) Results: Our results have shown that hemoglobin level upon diagnosis of CML has been the only hematologic parameter correlated to the risk of contracting COVID-19 in our CML patients. (4) Conclusions: TKI treatment did not negatively influence COVID-19 risk or the response to the vaccine in our patients. The safety profile of the currently approved COVID-19 vaccines was similar to that of the general population.

## 1. Introduction

Chronic myeloid leukemia (CML) is a common hematological malignancy, accounting for approximately 20% of total leukemia cases. The pathophysiological mechanism of CML is well understood: a t(9;22)(q34;q11) translocation, which results in a shortened chromosome 22, called the Philadelphia chromosome. Through this process, the ABL1 gene from chromosome 9 is juxtaposed with the BCR gene from chromosome 22, resulting in a BCR-ABL1 fusion gene that codes for BCR-ABL1 transcripts and fusion proteins with increased tyrosine kinase activity. Additional molecular mechanisms have also been discovered [1]. The life expectancy of CML patients is now close to the life expectancy of the general population for all ages with 5-year overall survival rates ranging 85–95% [2]. This success could be achieved with modern treatment options, such as tyrosine kinase inhibitors (TKIs). New diagnostic techniques also play a role in the significant improvement of outcomes [3]. Current guidelines recommend TKIs (imatinib, dasatinib, and nilotinib) as front line therapies [4]. Coronaviruses, members of the Coronaviridae family, are respiratory pathogens that infect bats and humans alike. When humans contract coronaviruses, they may exhibit no symptoms or symptoms such as fever, cough, or shortness of breath, but other (neurological, cardiovascular, or gastrointestinal) manifestations have also been reported. In certain cases, particularly in the elderly and among the immunocompromised, the infection can lead to severe systemic inflammatory response syndrome (SIRS) and, subsequently, death. Three major coronavirus outbreaks have been reported so far, with the most recent epidemic being the spread of the newly identified SARS-CoV-2, which is responsible for coronavirus disease-2019 (COVID-19). COVID-19 is an unprecedented healthcare crisis. The infection rate is 1–2% in the general population with a mortality rate of 1–5%. In these 2 years of the pandemic, the following groups have been identified as high risk: immunocompromised patients, the elderly, obese patients, patients with diabetes, and people of African American descent [5].

The prevalence of SARS-CoV-2 infection in CML patients is comparable to that of the overall population [6]. In the context of CML, there is no evidence that TKIs increase the risk of acquiring SARS-CoV-2 or causing severe COVID-19. However, some TKIs may cause side effects that are similar to COVID-19-induced organ damage or predispose patients to complications [7]. Severe COVID-19 could make treating these patients challenging. COVID-19 in the CANDID trial resulted in a 13.7% mortality rate among CML patients. Factors associated with a higher mortality rate were age and imatinib therapy. As of July 2020, 110 patients treated for CML were diagnosed with COVID-19. The patients from 20 countries had a median age of 54 years (18–89), and the median time from CML diagnosis was 7 years (0–25). Most patients were treated with TKIs (36% imatinib, 16% nilotinib, 11% dasatinib, 5% bosutinib, and 2% ponatinib) and 7% were in treatment free remission. Importantly, 45% patients had mild COVID-19 with no hospitalization required, and 17% had a severe infectious disease with a 14% mortality rate. 30% of patients interrupted TKI treatment during COVID-19 [8]. In the study published in August 2021 by Karki et al., there was a higher rate of mortality of 37% for patients with CML and COVID-19 [7]. The study showed an overall seroconversion rate in hematological patients of 46–85% after two full doses of SARS-CoV-2 vaccines [9].

Physicians should also be aware that even after two doses of the mRNA SARS-CoV-2 vaccine, many patients with hematologic malignancies are at risk of not producing antibodies. According to Greenberger, the seronegative rate following administration of a COVID-19 vaccine was 2.9% in patients with CML, 9% in patients with acute myeloid leukemia (AML), and 12% in patients with acute lymphoblastic leukemia (ALL). In comparison to BNT162b2 (BioNTech-Pfizer), patients were considerably more likely to develop an immune response to the mRNA-1273 (Moderna) vaccine series—odds ratio [OR] 1.50; 95% confidence interval (CI) 1.12–2.00; *p* = 0.007 [10].

The data regarding the risk of SARS-CoV-2 infection among cancer patients is still scarce, as well as for CML patients. However, case reports, such as the one reported from Abdalhadi et al. [11], show favorable evolution if the CML patients get infected under TKI treatment. Li et al. also performed a study by phone or electronic questionnaires. The study included 530 CML patients from 29 centers in Hubei Province. Among them, the prevalence of COVID-19 was 0.9%. The disease had a mild evolution with one death. The authors identified comorbidities and no complete hematologic remission. In addition, advanced stage CML at diagnosis has been identified as an individual risk factor for COVID-19 [12]. The outcome of COVID-19 seen in CML patients could be explained by the immune restoration under TKI treatment and the favorable evolution of CML. Moreover, some TKIs, such as imatinib, were shown to have in vitro antiviral activity against MERS and SARS-CoV2 [13,14,15]. 

Case reports regarding CML and COVID-19 infection or vaccination have also been published in the recent years (Table 1). Considering these, and based on our long experience with CML treatment [16,17], the need for more comprehensive and up-to-date information regarding CML and COVID-19 is evident. Our study aims to document the experience we gained with COVID-19 in a group of CML patients treated at the Ion Chiricuta Cancer Center’s hematology department, in Cluj-Napoca, Romania. Additionally, we report the COVID-19 vaccination rate and adverse events of the vaccine in this cohort. Our study also investigates whether hematological parameters are associated with COVID-19 infection severity in CML patients.

## 2. Materials and Methods

### 2.1. Patient Demographics

The study included CML patients diagnosed at the Ion Chiricuta Cancer Center from 1996 to 2020. Inclusion criteria were age older than 18 years, survival at the time of study, and treatment with TKIs. Exclusion criteria included death occurring before the trial began, persistent hospitalization, discontinuation of the TKI therapy except for the SARS-Cov-2 infection period, and accidentally positive SARS-CoV-2 RT-PCR results. To determine if patients met the eligibility criteria, medical records of 286 individuals were examined. A team of healthcare professionals, including clinicians and pharmacists, conducted phone interviews. All patients included in the study provided informed consent. The interview included a questionnaire divided into three sections: general health and COVID-19 infection, immunization status, and adverse effects of the COVID-19 vaccine. Table 2 summarizes patients’ demographics. 

During 2020, our center followed the European guidelines for SARS-CoV-2 testing for hematologic patients: all unvaccinated patients; those within 180 days from a COVID-19 infection; within 48 h for all asymptomatic patients before chemotherapy, radiotherapy, surgery and hospitalization, immunosuppression therapy; or immunocompromised patients. 

### 2.2. Hematologic Parameters and Response Assessment 

We are using the European LeukemiaNet (ELN) criteria to assess response to TKI treatment [28]. As such, complete hematologic response (CHR) means an asymptomatic patient with no palpable spleen and a normal blood count with a white blood count of <10 × 10^3^/microliter, no immature cells, basophils <5%, and platelets <350 × 10^3^/microliter. The complete cytogenetic response (CcyR) corresponds to no Ph+ metaphases by chromosome banding and <1% (≤1/200) nuclei by FISH. Complete molecular response (CMR) corresponds to undetectable BCR-ABL transcript and major molecular response (MMR) is equal to BCR-ABL/ABL ≤0.1%. Hematologic monitoring was performed by a general clinical exam, complete blood count, and peripheral smear. Molecular monitoring was done by RQ-PCR using a LightCycler platform: 10–1,000,000. The method quantifies the b2a2 and b3a2 fusion transcripts corresponding to the P210 protein.

### 2.3. COVID-19 Diagnosis

RT-PCR for SARS-CoV-2 was performed using nasopharyngeal swab samples collected on a viral transport medium (VTM), followed by an automated extraction using Miracle-AutoXT (Intron Biotechnology), then real time PCR amplification using an IVD kit. The technique using multiplex PCR identifies three target genes for the specific detection of SARS-CoV-2 and an internal control. The limit of detection has been 15 RNA copies/reaction.

### 2.4. Statistical Analysis

Study data were collected and analyzed using R 3.5.1 software [29]. In the final analysis, all participants for whom the variables of interest were available have been included. No assumptions were made to account for missing data. Data have been presented as counts, percentages, and medians. The Fisher test was used for contingent tables. Standard normal distribution was obtained using Shapiro–Wilk’s test. A *p* value under 0.05 was considered statistically significant. 

## 3. Results

There are 289 CML patients being treated in our department. Data for these patients diagnosed between 1996 and 2020 and treated with TKIs have been analyzed. Unfortunately, according to current reluctance, only 51 patients agreed to give a phone interview regarding their COVID-19 and their vaccination status. Thus, informed consent has been obtained from these 51 patients, out of which 28 (54.9%) were male and 23 (45.1%) were female. Among these, only 10 patients reported COVID-19 infection, which is in line with already published data comprising multiple centers. The median age at diagnosis for the included cohort was 48 years. 

Risk stratification of CML survival has been done using the SOKAL and EUTOS scoring systems: 32 (62.7%) patients had a low SOKAL score, 15 (29.4%) had an intermediate score, and 2 (3.9%) had a high SOKAL score, whereas 41 (80.3%) had a low EUTOS score.

Each patient received a TKI as front line treatment. The 2 most common TKIs prescribed were imatinib (74.5%) and dasatinib (49%). Other TKIs used were nilotinib (41.2%), ponatinib (7.8%), and bosutinib (5.9%). Table 3 summarizes TKIs prescribed for the patients included in the study.

Major molecular response (MMR) was obtained in 43 (84.3%) patients, in 19 patients following 1 line of TKI (11 imatinib, 6 dasatinib, 2 nilotinib), in 8 patients after 2 lines of TKI, in 3 patients following 3 lines of TKI, and 1 patient obtained MMR after 4 lines of TKI. Complete molecular response (CMR) has been obtained after 1 line of TKI in 10 patients (8 imatinib, 2 dasatinib) and following 2 lines of treatment in 3 patients. 

TKI treatment was well tolerated, and the most frequently reported adverse event was fatigue. Patients treated with imatinib complained of nausea, vomiting, muscular cramps, peripheral edema, and diarrhea. Treatment with antiemetics and ion supplementation was effective in all cases. No interruptions have been reported. Patients treated with nilotinib reported rash and pancreatic enzymes elevation, hyperglycemia, and increased bilirubin without jaundice. No drug interruptions or dose reductions were needed. Antihistamines were allowed as concomitant medication. Among patients treated with dasatinib, about 20% presented with pleural effusion, grad 1 or 2, that was treated with corticosteroids, pleural drainage, and temporary interruption of the TKI. During bosutinib treatment, patients complained of diarrhea, even at a lower dose of the target dose of 500 mg daily, but it was managed with diet and loperamide treatment. Ponatinib was well tolerated, but strict cardiovascular monitoring was performed.

TKI treatment was monitored according to European Leukemia Net (ELN) guidelines, with clinical and hematologic exams every month and BCR-ABL1 transcript quantification every 3 months until major molecular response. Moreover, the criteria of response were established according to ELN guidelines.

In ten cases, COVID-19 has been diagnosed by RT-PCR. These patients tested positive in February and May of 2021. All CML patients that tested positive for SARS-CoV2 were in MMR, under TKI treatment. Among COVID-19 patients, 30% discontinued TKI treatment during COVID-19. The hematologic parameters (at diagnosis of CML) of these patients have been summarized in Table 4. The results of the statistical analysis of possible risk variables for COVID-19 infection in CML patients is shown in Table 5. Among the 10 COVID-19 patients, 7 patients were not vaccinated. Most patients had a mild case of COVID-19 with symptoms consisting of fever as the most frequent symptom, in addition to rhinorrhea, cough, headache, muscle pain, weakness, and diarrhea. Only 2 patients were hospitalized (1 patient was vaccinated, and 1 patient was not vaccinated). During hospitalization, all drugs indicated by our infectious disease specialists were monitored for TKI interactions. Three patients interrupted TKI treatment during the course of COVID-19. The hospitalized patients presented with respiratory failure and hyperpyrexia. There have been no ICU admissions, long term hospitalizations, or deaths reported due to COVID-19 in our CML cohort.

Out of the 51 patients included in the study, 24 patients received SARS-CoV-2 vaccination from January to August 2021. Data regarding COVID-19 infection and vaccination status have been summarized in Table 6. Out of the 24 patients, 19 received BioNTech-Pfizer vaccine, 2 patients received Astra Zeneca, and 3 patients received Johnson and Johnson. In total, 7 vaccinated individuals experienced adverse events: 3 experienced local discomfort, 3 experienced fever and myalgia, and 1 experienced gastrointestinal symptoms. There has been no statistically significant link shown between vaccination adverse effects and hematologic parameters at the time of diagnosis or TKI therapy. The statistical study of possible risk variables for adverse effects following COVID-19 immunization in CML patients is summarized in Table 7.

## 4. Discussion and Conclusions

Our study aimed to investigate if hematologic parameters or TKI therapy increase the risk of SARS-CoV-2 infection in CML patients. The only variable associated with COVID-19 risk was hemoglobin level upon CML diagnosis. TKI treatment did not negatively influence the COVID-19 risk or response to vaccine in our patients. 

A possible explanation is that the target of TKIs used in CML is not limited to the BCR-ABL fusion protein. The first TKI used was imatinib, which also inhibits c-ABL, c-kit, ARG (ABL related gene), and PDGFR (platelet-derived growth factor receptor) kinases. These properties suggest a role in attenuating inflammation and the vascular leak syndrome [30]. Imatinib also has antiviral properties against SARS-CoV2 and MERS-CoV, as demonstrated in pre-clinical studies [14,31]. The multitargeted second generation TKI, dasatinib, inhibits BCR-ABL protein, c-kit, PDGFR, SRC family, DDR1, and 2 (collagen discoidin domain receptor 1 and 2), leading to a reduction in inflammation [32]. Both dasatinib and bosutinib showed anti-inflammatory properties by inhibition of salt-inducible kinases [33].

The COVID-19 immunization rate in Romania by September 2021 has been 27.5% for the general population and 47% for our CML patient group. The Pfizer vaccine has been available in our country since January 2021, Moderna and AstraZeneca vaccines have been available since February 2021, and Johnson & Johnson has been available since May 2021. According to the National Institute of Public Health and the National Committee for COVID-19 vaccination, by September 22nd 2021 (World CML Day), a total of 5,286,623 individuals received complete schema vaccination against SARS-CoV-2. There were 1821 total local adverse events and 15,305 systemic adverse events reported for the vaccines. A statistical report of the same day (September 22nd 2021) reported a total number of 1,769,783 coronavirus cases in Romania, with 55,617 deaths and 1,658,933 recovered patients. Our results show that the vaccine’s safety profile for our CML patients is comparable to that of the general population. The National Comprehensive Cancer Network (NCCN), the European Society for Medical Oncology (ESMO), local guidelines, and our institution further recommend administration of the COVID-19 vaccine to CML patients. NCCN also recommends that all patients with active hematologic malignancies should receive the third dose of COVID-19 vaccine as well. Data presented at our National Hematology Conference, unpublished, showed a 50% rate of SARS-CoV-2 vaccination for CML patients in another hematology center, the Coltea Hospital. In a CML cohort from Colentina Hospital, four CML patients needed hospitalization for COVID-19. The patients were under ponatinib and nilotinib treatment. Their COVID-19 disease was mild to moderate. During COVID-19, the patients were treated with off-label arbidol, remdesivir, and heparin. Even after almost 3 years of the pandemic, there is limited information on several aspects regarding CML and COVID19. Alongside the immense efforts made by the medical staff and the scientific community since 2020, more case reports and multicenter studies have been published and discussed in the introduction. Thus, data regarding COVID-19 and CML are becoming clearer. Our small study confirms the findings published recently, and our goal is to propose to the Romanian Society of Hematology that TKI treatment should be continued during COVID-19, with special attention regarding possible drug-to-drug interactions. Because of the small cohort size, we cannot draw specific conclusions; instead, we confirm the findings of other centers’ case reports. Further investigation is needed regarding the following aspects: (1) the risk of contracting SARS-CoV-2 for patients with hematological malignancy; (2) the differences in the clinical picture of the infection in CML patients; (3) the outcome of COVID19 in CML patients; and (4) the incidence of thrombosis in CML patients suffering from COVID-19. For the entire cohort of 286 patients treated at our center, 79.9% of patients obtained major and complete molecular response under TKI treatment, and 20.1% patients had only a hematological response or progression.

Even after 2 years of the pandemic, there is still a reluctance in declaring COVID-19 infection, and the rate of vaccination is still very low especially among our immunocompromised patients, even after intensive governmental campaigns for immunization. With the immense work of the medical staff around the world, however, information is continuously gathered. There are also data reported on other TKIs and their action in COVID-19 infection. Since the beginning of the SARS-CoV-2 pandemic, it was noted that severe COVID-19 cases were associated with an overwhelming inflammatory reaction/ macrophage activation syndrome with increased levels of cytokines like IL2, IL7, IL10, GSCF (granulocyte colony-stimulating factor), IP10 (human interferon-inducible protein 10), MCP1 (monocyte chemoattractant protein 1), MIP1A (macrophage inflammatory protein 1A), or TNFα, meaning a true “cytokine storm” associated with inferior prognostic. Considering the lack of specific anti-SARS-CoV-2 medication and the cytokine storm complication, as well as the experience with graft versus host disease refractory to corticosteroids [34], trials using ruxolitinib were started. Ruxolitinib is a tyrosine kinase inhibitor selective for JAK1 and JAK2 (Janus kinase 1 and 2), used in the treatment of myeloproliferative disorders, such as policythemia vera and primary myelofibrosis. In a randomized trial of 53 COVID-19 patients treated with standard-of-care drugs (antivirals, preventive antibiotics, corticosteroids, positive pressure ventilation, renal filtration, mechanical ventilation and vasoactive support), Yang and co-authors showed that patients treated with ruxolitinib 5 mg BID had a faster recovery than the control arm. Although the difference was not statistically significant, there were no deaths in this group, compared with a 14.3% death rate in the control arm at 28 days [35]. Another case presentation showed favorable evolution in a tocilizumab refractory case of COVID-19, with ruxolitinib 10 mg BID and no side effects [36]. Another TKI used for Rheumatoid Polyarthritis is baricitinib [37]. In four studies including 10,815 COVID-19 patients, favorable outcome was observed in the baricitinib arm compared with standard therapy [38]. In addition, more selective anti-JAK1 inhibitors that inhibit T lymphocytes are being studied for both infectious and malignant diseases. For the future, as knowledge of the pathology and treatment for COVID-19 evolves, information on drug-to-drug interactions, on treatments’ adverse events, on disease outcome, and on the outcome of comorbidities is more and more needed.

## 5. Limitations

There are several limitations to this study. Our study was conducted on an uncontrolled cohort, so prevalence of findings cannot be compared with patients without COVID-19. Furthermore, many patients declined to participate in the telephone interviews. As a result, only a small number of patients are included, and patients who did not participate may have experienced fewer symptoms than those who did. Another limitation to our study is the lack of antibody evaluation because our study was performed by phone interviews. Moreover, there are few laboratories that perform these tests in Romania. In a Romanian study, published in April 2022, Vata et al. showed high specificity for IgA and IgG antibodies following seroconversion, but the time frame of their positivity is still unknown [39]. It is also unclear if this detection is of clinical value. We agree with our colleagues that antibody detection could be of value for asymptomatic patients or patients with a low PCR viral load or in the case of long COVID-19.

## Figures and Tables

**Table 1:** Recent articles on CML and COVID-19 or COVID-19 vaccination.

CML Associated with	Article
COVID-19	Yılmaz et al. [18]
	Graf et al. [19]
	Asif et al. [20]
	Ali et al. [21]
	Delgado et al. [22]
	Claudiani et al. [23]
	Karki et al. [7]
	Rea et al. [8]
	Breccia et al. [24]
COVID-19 vaccination	Kuan et al. [25]
	Claudiani et al. [26]
	Harrington et al. [27]
	Greenberger et al. [10]

**Table 2:** Patient demographics.

Patients Included (%)		51 (100)
Gender	Female	23 (45.1)
	Male	28 (54.9)
Age at Diagnosis (median)		48 (37–59)
Age at Assessment (median)		53 (46–66)

**Table 3:** TKIs prescribed for the 51 patients included in the study (some patients needed multiple lines of treatment).

**TKI Treatment (%)**	Imatinib	38 (74.5)
	Dasatinib	25 (49)
	Nilotinib	21 (41.2)
	Ponatinib	4 (7.8)
	Bosutinib	3 (5.9)

**Table 4:** Hematologic parameters of the patients included in the study.

Hematologic Parameter	Value
white blood cells (×10^9^/L)	99 (39–176)
hemoglobin (g/dL)	11 (10–12)
platelets (×10^3^)	294 (237–503)
peripheral blasts (%)	1 (0–3)
basophils (%)	4 (2–6)
eosinophils (%)	1 (0–3)
LDH (IU/L)	1000 (483, 2031)

**Table 5:** Statistical analysis of possible risk variables for COVID-19 infection in CML patients.

	COVID-19 Diagnosed		*p*-Value
	YES (*n* = 10)	NO (*n* = 40)	
Female	4 (40%)	19 (48%)	0.736
age at diagnosis	43 (37–60)	48 (37–58)	0.941
age at assessment	51 (40–67)	52 (47–65)	0.941
white blood cells (×10^9^/L)	73 (48–180)	102 (34–170)	0.845
hemoglobin (g/dL)	12 (11–13)	11 (10–12)	**0.044**
platelets (×10^3^)	2 (2–3)	297 (239–510)	0.389
peripheral blasts	2 (2–3)	1 (0–3)	0.198
basophils	4 (3–6)	4 (1–6)	0.809
eosinophils	1 (0–2)	1 (1–3)	0.697
LDH	935 (553–1570)	1000 (430–2000)	0.947
imatinib	7 (70%)	31 (78%)	0.686
nilotinib	2 (20%)	19 (48%)	0.160
dasatinib	4 (40%)	20 (50%)	0.728
bosutinib	0 (0 %)	4 (10%)	0.571

**Table 6:** Data regarding COVID-19 of the 51 patients included in the study.

**COVID-19 infection until August 2021 (%)**	10 (19.6)
**COVID-19 vaccination status until August 2021 (%)**	24 (47.1)
**COVID-19 vaccine adverse effects (%)**	7 (31.8)

**Table 7:** Statistical analysis of possible risk variables for adverse effects following COVID-19 immunization.

	COVID-19 Diagnosed		*p*-Value
	YES (*n* = 7)	NO (*n* = 15)	
female	3 (42.9%)	19 (46.7%)	0.125
age at diagnosis	42 (32–50)	52 (40–61)	0.145
age at assessment	48 (42–62)	60 (51–71)	0.941
white blood cells (×10^9^/L)	100 (26–155)	129 (63–200)	0.588
hemoglobin (g/dL)	12 (11–14)	11 (10–13)	0.164
platelets (×10^3^)	324 (243–410)	286 (268–303)	0.699
peripheral blasts	0 (0–1)	1 (0–3)	0.403
basophils	3 (3–8)	3 (1–6)	0.603
eosinophils	2 (1–2)	0 (0–1)	0.329
LDH	1000 (455–2069)	600 (200–1500)	0.751
imatinib	7 (100%)	12 (80%)	0.522
nilotinib	1 (14%)	9 (60%)	**0.074**
dasatinib	4 (57%)	9 (60%)	1
bosutinib	0 (0 %)	2 (13%)	1

## Data Availability

The data have been generated based on the results of the patients treated in our clinic and these data are not public because of patient confidentiality.
